# Subcutaneous nodules and flexion contractures of the hands

**DOI:** 10.1016/j.jdcr.2025.05.050

**Published:** 2025-07-10

**Authors:** Emily J. Levin, Ben J. Friedman, Marsha Chaffins, Natalie H. Matthews

**Affiliations:** a UPMC Department of Dermatology, School of Medicine, University of Pittsburgh, Pittsburgh, Pennsylvania; b Department of Dermatology, Henry Ford Health System, Detroit, Michigan; c Department of Dermatology, Wayne State University, Detroit, Michigan; d Department of Medicine, Michigan State University College of Human Medicine, East Lansing, Michigan

**Keywords:** cutaneous nodules, dermatoarthropathy, fibroblastic rheumatism, fibrosing dermatosis

## History

**Fig 1:**
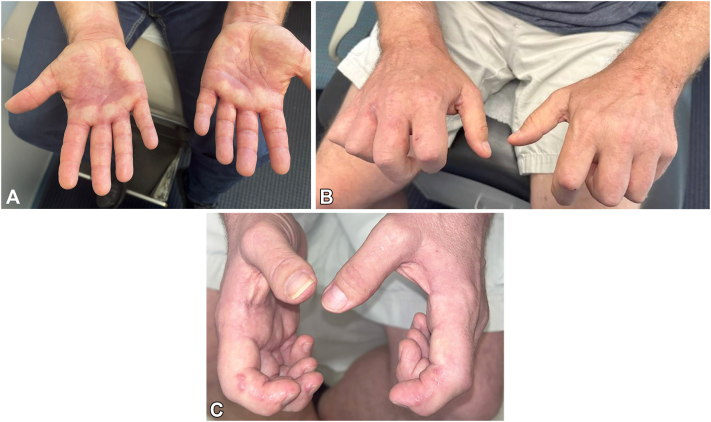
**A,** Clinical appearance of fibroblastic rheumatism-associated finger flexion contractures. Flexion contracture of both hands, left hand more severe than right, demonstrating limited finger mobility. **B,** Clinical appearance of fibroblastic rheumatism-associated finger nodules. **C,** Red-brown 5 mm nodules are seen on the radial aspects of the second and third fingers of the right hand with a linear-like distribution reminiscent of coral beading.

**Fig 2:**
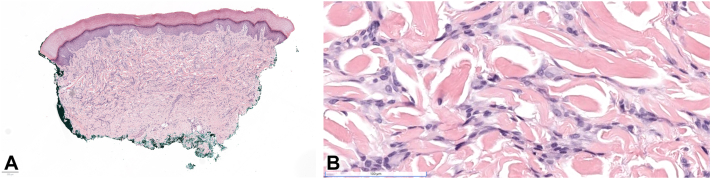
Histopathology of fibroblastic rheumatism. A punch biopsy from a nodule on the second finger demonstrates a dermal based spindled cell proliferation within a heavily collagenized background on acral skin under (Hematoxylin-eosin, original magnification). **A,** low magnification, and **B,** high magnification.

A 50-year-old male presented with nodules on his hands and painful, limited finger mobility. He reported that 9 months prior to presentation, he developed abrupt purple discoloration of the palms, progressing to severe flexion contractures of both hands (Fig 1, *A* and *B*). Two months prior, he developed painless red-brown 3-5 mm nodules distributed linearly and symmetrically on the lateral aspects of his fingers bilaterally (Fig 1, *C*). His personal and family history were unremarkable. Autoimmune workup revealed positive SSA and weakly positive CCP, while other serologies—including ANA, RF, dsDNA, complement levels, anti-Sm, anti-RNP, anti-SCL70, anti-Jo-1, anti-SSB, and ACA—were negative. Infectious work-up was negative including hepatitis B and C. X-ray and magnetic resonance imaging revealed mild degenerative changes, nonspecific bone demineralization, and flexor tenosynovitis with joint erosion in the hands. Punch biopsies of the finger nodules revealed dermal fibrosis with spindle-fibroblastic cell proliferation, increased collagen, and decreased elastic fibers (Fig 2, *A* and *B*). Immunohistochemical staining was positive for CD4 and CD14, and negative for CD68, CD163, LCA, and α-SMA. He was treated with oral corticosteroids, methotrexate, adalimumab, and physical therapy. Methotrexate and oral steroids improved pain, resolved the cutaneous nodules, and slowed progression. It did not restore his joint damage and finger mobility.

**Question: What is the diagnosis?**

**A.** Multicentric reticulohistiocytosis
**B.** Fibroblastic rheumatism (FR)
**C.** Rheumatoid arthritis
**D.** Nodular scleroderma
**E.** Granuloma annulare

**Answer**

**B.** Fibroblastic rheumatism (FR) – Correct. FR is a rare, rapidly progressive dermato-arthropathy characterized by 2-20 mm cutaneous nodules commonly found around affected finger joints, and symmetric polyarthritis.^1^^,^^2^ It affects all ages and genders^1^ and has unclear etiology with some suggesting a fibroblastic response to unidentified triggers including infection.^2^^,^^3^

Histopathologically, it is characterized by a dermal-based fibroblastic and myofibroblastic spindle cell proliferation set within a collagenous stroma. Decreased elastic fibers have been reported.^4^ Immunohistochemistry is variable, with some cases being reported to label with α-SMA and vimentin.^1^ Laboratory tests for autoimmune antibodies, including ANAs, RF, anti-dsDNA, anti-CCP, anti-Smith, anti-SSA, anti-SSB, anti-Scl-70, anti-Jo-1, CRP, and C3 are typically negative.^2^

It can be clinically challenging to distinguish FR from MRH, as both can present with red-brown nodules clustered over joints of the hands with rapidly destructive symmetric polyarthritis,^5^ however their histopathology is distinct. Due to the rapid progressive nature of FR, timely treatment is critical. High-potency systemic corticosteroids, methotrexate, adalimumab, α-interferon, colchicine, hydroxychloroquine, nonsteroidal anti-inflammatory drugs, and physical therapy have been tried as treatment with variable success. Moderate-to-high doses of systemic steroids may be beneficial in inhibiting fibrogenic cytokine production and subsequent fibroblast activation, which can benefit arthritis symptoms in early active stages of FR.^1^^,^^3^ However, treatment rarely resolves symptoms completely or prevents joint damage. We urge clinicians to include FR in the differential when patients present with hand-limited cutaneous nodules and polyarthritis, as timely treatment is critical to prevent irreversible damage.

## Conflicts of interest

None disclosed.
